# Linking Neural and Symbolic Representation and Processing of Conceptual Structures

**DOI:** 10.3389/fpsyg.2017.01297

**Published:** 2017-08-10

**Authors:** Frank van der Velde, Jamie Forth, Deniece S. Nazareth, Geraint A. Wiggins

**Affiliations:** ^1^Department of Cognitive Psychology and Ergonomics, Faculty of Behavioural, Management and Social sciences, University of Twente Enschede, Netherlands; ^2^Institute of Psychology (IOP) Leiden, Netherlands; ^3^Department of Computing, Goldsmiths University of London London, United Kingdom; ^4^Computational Creativity Lab, Cognitive Science Group, School of Electronic Engineering and Computer Science, Queen Mary University of London London, United Kingdom

**Keywords:** cognitive architecture, memory representation, hebbian learning, compositional learning, incremental learning, *In situ* representations

## Abstract

We compare and discuss representations in two cognitive architectures aimed at representing and processing complex conceptual (sentence-like) structures. First is the Neural Blackboard Architecture (NBA), which aims to account for representation and processing of complex and combinatorial conceptual structures in the brain. Second is IDyOT (Information Dynamics of Thinking), which derives sentence-like structures by learning statistical sequential regularities over a suitable corpus. Although IDyOT is designed at a level more abstract than the neural, so it is a model of cognitive function, rather than neural processing, there are strong similarities between the composite structures developed in IDyOT and the NBA. We hypothesize that these similarities form the basis of a combined architecture in which the individual strengths of each architecture are integrated. We outline and discuss the characteristics of this combined architecture, emphasizing the representation and processing of conceptual structures.

## 1. Introduction

The ability to represent and process conceptual structures, as found in language processing, reasoning, and in generating conceptual representations from visual and auditory perception, are key elements of human cognition. They can be studied with the aim to understand human cognition and its relation to the brain. But they can also be targets for the development of artificial cognitive systems. These aims can be combined to various degrees, because a cognitive architecture that provides an understanding of a (neural) cognitive process can also be used in artificial systems, and, conversely, the way in which an artificial system processes complex information can reveal aspects of human processing as well.

Here, we discuss and relate the different representations used in two cognitive architectures, one neural and one symbolic, in which complex conceptual structures can be represented and processed. That is, we discuss and illustrate the different ways in which complex conceptual structures are represented or learned in the two architectures and how these representations could be related.

In particular, we aim to outline how combined representations could be developed, for use in a combined architecture in which aspects of our neural and symbolic architectures are integrated. We hypothesize that such a combined architecture could serve as a model of human conceptual processing and its relation to the brain. When implemented, it could also serve as a new artificial architecture in which forms of neural (parallel) hardware and neural and symbolic forms of learning and processing could be integrated. We are as yet at the beginning of the integration of our architectures, which is also a reason why we focus on issues of representation here.

The neural representation in our integration is that used in the Neural Blackboard Architecture (NBA), which is aimed to represent and process conceptual structures in language (e.g., van der Velde and de Kamps, 2006, 2010), reasoning and other cognitive domains (van der Velde, 2016a). The NBA assumes that conceptual representations in the brain consist of dedicated network structures, or neural assemblies, that develop over time and that can be distributed over wide areas in the brain and cortex. A fundamental characteristic of these network-like conceptual representations is that they are always content addressable, whether they are activated in isolation or whether they are parts of more complex (and even hierarchical) conceptual structures, such as sentences in language.

The NBA provides “neural blackboards” that afford the representation and processing of complex conceptual structures based on neural assembly conceptual representations in specific cognitive domains. Examples are neural blackboards for sentence structures, phonological structures, sequences, and relations as used in reasoning. In each domain, a dedicated neural blackboard will provide a range of specialized structural elements that can bind in a neural manner to the neural assemblies (e.g., representing “words” in language). The neural bindings, implemented with neural circuits, allow the creation and processing of more complex cognitive structures (e.g., “sentences”) in a combinatorial manner.

The symbolic representation in our integration is that used in Information Dynamics of Thinking (IDyOT). IDyOT derives (e.g., sentence-like) structures by learning statistical sequential regularities over a linguistic corpus (Wiggins, 2012b; Wiggins and Forth, 2015; Forth et al., 2016). IDyOT is unusual as a machine learning formalism in that it is symbolic in nature, but it generates and gives explicit semantics to its own symbols, in a bottom-up learning process, which is optimized by a general, data-independent principle of information efficiency, conceptualized as predictive accuracy. These symbols correspond with concepts in the semantics of the system. Another unusual aspect of IDyOT's operation is that both representations and sequential models are optimized simultaneously with respect to the prediction accuracy of the models, causing a trade-off between overfitting and accuracy that we propose as a model of the corresponding trade-off in human cognition. The explanation of this process is a novel contribution of the current paper.

The representational links between IDyOT and NBA concern the nature of the dedicated structural elements that allow processing and representation of complex conceptual structures, the way these elements could be activated during processing, and the underlying semantics of the architectures in the form of conceptual spaces that possess a geometrical structure (Gärdenfors, 2000, 2014).

In the NBA, the dedicated structural elements form the neural blackboards. The kinds of elements used and the way they are activated derive from analyses of the cognitive domains at hand, as in the sentence NBA (e.g., van der Velde and de Kamps, 2006, 2010). However, the combination of NBA with IDyOT provides the possibility to derive these structural elements by learning from real corpora. Conversely, the NBA could provide a neural implementation of the more higher-level formal account as provided by IDyOT. Thus, IDyOT potentially supplies a higher-level formal account and learning abilities to the operations of the NBA. Conversely, the NBA provides a route toward a neural implementation of IDyOT, which could also form the basis of in parallel operating hardware.

## 2. Theoretical position and novelty

Our theoretical position here is that the representations used in NBA and IDyOT are in fact two different representations of the same thing, at different levels of abstraction, but with focus on similar representational affordances. In the following sections, we describe the representations, and the relations between them—but, as always, to understand the representations it is necessary also to understand the processes that work over them.

The novelty in the current paper lies in several places, primarily in the thorough-going comparison between the representations and corresponding processes in the two architectures. The entire description of IDyOT memory construction is also novel, and we present a novel simulation of neural activity based on the NBA, which allows for a detailed comparison with brain activity observed in human (sentence) processing. To the best of our knowledge, such a detailed potential comparison between human brain activity and simulated model activity is not available in the case of high-level cognitive processing, such as sentence comprehension. This also strongly motivates the integration of our architectures, because that would endow the NBA with the learning capabilities of IDyOT, based on real corpora (as outlined below). In turn, the dynamics and structure of the NBA would then allow a comparison between the representations and underlying processing as learned by IDyOT and human brain activity.

The structure of the paper is as follows. In the next two sections we briefly describe the representations used in NBA and IDyOT in turn, also giving detail of processing where appropriate. In the sections that follow, we discuss a number of specific links between NBA and IDyOT and the potential benefits of their integration.

## 3. Neural blackboard architecture

In our outline of Neural Blackboard Architecture, or NBA for short, we focus on the representation of concepts (e.g., underlying words) in the architecture and the representational structures that are used to integrate concepts in more complex cognitive structures, such as relations and sentences.

The basis of concept representation in the NBA are “neural assemblies,” as proposed by Donald Hebb (1949). In the view of Hebb, these neural assemblies develop over time by interconnecting the neurons in the brain that are involved in processing (sensory) information and generating actions related to the concept they represent. However, unlike Hebbian assemblies, conceptual representations in the NBA are not only associative. Instead, they can (and mostly will) contain relational structures as well.

**Figure 1:**
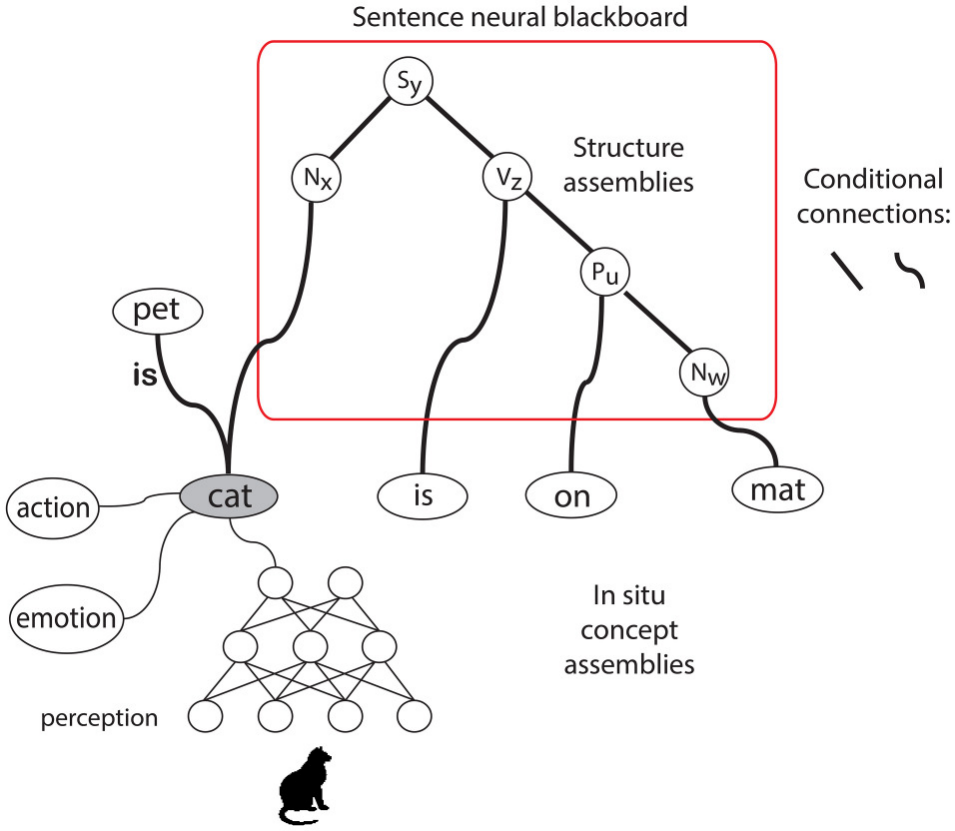
**Left**: Neural assembly representation of *cat*. **Right**: Sentence structure in the Neural Blackboard Architecture: a sentence neural blackboard (temporarily) interconnects the “*in situ*” concept representations (given by neural assemblies) of *cat, is, on*, and *mat* to form the sentence structure *cat is on mat*. The thick line connections represent “conditional connections.” They can be opened by gating circuits that are either activated by sustained activity in working memory neural populations (representing binding) or by neural control circuits (e.g., performing parsing operations). N, noun; P, preposition; S, sentence; V, verb.

Figure 1 illustrates a neural assembly representation of *cat*. It would be distributed over different areas in the cortex and brain, depending on the kind of information involved, including networks processing perceptual information about cats and networks that can produce specific actions (e.g., pronouncing the word “cat”). But also networks representing emotional content or associations related to cats belong to the assembly, and networks that instantiate relations, such as *cat is pet*.

The combination of perception and action in the assembly structure of a concept entails that both the patterns (and activation) of “incoming” and “outgoing” connections determine the meaning of a concept. For example, neurons observed in the medial temporal cortex responded to a person whether the person or his or her name was presented (Quian Quiroga, 2012). These “perception” networks do in fact belong to the assembly structure of a concept, because without them the concept could not be activated (or was not learned). But this would capture only part of the role and (hence) meaning of the conceptual representation involved. Equally important would be the effect of these neurons on downstream processing (van der Velde, 2015).

The notion that conceptual representations interconnect sensory information processing and action generation underscores their role in producing behavior. The ability to produce behavior is a crucial aspect of cognition (and hence of every neuro-cognitive model) because the evolution of cognition depended on the ability to produce behavior. Without advocating a behavioristic view of cognition (e.g., as the basis of modeling cognition) we do argue that the prime role of cognition is to intervene in the reflex (cf. Shanahan, 2010). In this view, the need for a connection structure that transfers sensory activity to motor activity should always be at the background of a neural-cognitive model.

So, in Figure 1, it is not just the gray oval that represents the concept *cat*, but instead the entire network structure to which it is connected. The gray oval could play the role of a higher-level representation of the concept in the sense that it interconnects the concept to other networks. But it would be wrong to see this as the “genuine” encoding of the concept. Without the networks to which it is connected, the gray oval does not encode anything.

An important feature of conceptual representations given by neural assemblies is that they are “*in situ*.” This entails that they cannot be copied and transported to create more complex structural representations with them (e.g., as found in language or reasoning). Instead, the same assembly (or a part thereof) is always activated when the concept it represents is tokened. One consequence of this kind of representation is that an assembly can develop and grow over time, as originally discussed by Hebb.

Another direct consequence of the *in situ* nature of a neural assembly is that the concept it represents is content addressable. This entails that the same assembly (or part thereof) will be activated when sufficient information about the concept it represents is available (e.g., perceived), even when the concept is part of a (complex) sentence structure.

Huth et al. (2016) give an indication of the *in situ* nature of conceptual representation in the brain. They measured brain activity related to words when people were listening to stories (in an fMRI scanner). The parts of the cortex that responded to the words (after statistical analysis) were much larger compared to previous studies in which only individual words were presented. The analysis divided the left hemisphere (LH) into 192 distinct functional areas, 77 of which were semantically selective. The right hemisphere was divided into 128 functional areas, 63 of which were semantically selective (even though the RH is usually regarded as not being involved in language). Remarkably, the organization of these areas was quite similar over the different (7) subjects involved in the study. Furthermore, next to these semantic areas, other areas also responded to other aspects of words (e.g., Broca's area).

Because the study was focused on semantic representation, the words observed in the study were categorized into 12 semantic domains. These domains tiled the cortex in terms of the 77 areas in the LH and the 63 areas in the RH referred to above. Inspection of the data reveals that semantic domains are generally represented in different tiles, distributed over the LH and/or RH cortex.

The semantic representation as observed by Huth et al. (2016) seems to be in line with the Hebbian assembly hypothesis, in that these representations would have arisen over time, and would (partly) be determined by the context in which the concepts were processed. This could explain why, e.g., the same visual concept (e.g., *colored*) activates areas near the visual cortex but also in the prefrontal cortex. This pattern of activation could reflect different parts of the assembly of the concept, and their selective activation would then be determined by the context (visual processing vs. motor behavior) in which the concept is used and learned. The fact that a concept generates activation in different cortical areas is in line with the assembly representation as illustrated in Figure 1.

### 3.1. Neural blackboards as connection paths

If concepts are represented and distributed as *in situ* assemblies, the question arises of how they could be combined to represent more complex cognitive structures, such as relations or sentences.

The key notion of the NBA is that more complex cognitive structures are formed by providing (temporal) *connection paths* between the assemblies (concepts) they contain, in relation with the structure they express. These (temporal) connection paths are formed and controlled in “neural blackboards.”

For example, in the case of language, the NBA provides a connection structure (or connection path) that allows arbitrary words in a given language to be (temporarily) interconnected in accordance with the structure of the sentence. The words in this case are the network structures (neural assemblies) as described by Huth et al. (2016). The neural blackboards in the NBA provide a “small world” network structure that would allow the *in situ* and distributed concept assemblies (“words”) to be interconnected using a limited set of intermediary “hubs and sub-hubs,” given by the structure assemblies and their potential bindings in the blackboards. Small world networks are found in a wide variety of natural and man-made structures because they allow arbitrary interconnectivity with minimal means. They also play an important role in the brain (Shanahan, 2010).

Figure 1 illustrates how in the NBA a sentence can be formed with *in situ* concepts encoded by neural assemblies. The *in situ* assemblies for *cat, is, on*, and *mat* are bound to a “neural blackboard” to form the sentence *cat is on mat*.

Figure 1 illustrates the very basic aspects of the neural blackboards that the NBA uses to encode relations between *in situ* concept assemblies. In the case of language there are (at least) two neural blackboards involved. One is a phonological blackboard, which is not illustrated here. The other is the sentence blackboard which encodes sentence structures, as illustrated here with the sentence *cat is on mat*.

The need for both a phonological and a sentence blackboard derives from the productivity of natural language. Language has (at least) a two tier productive structure (Jackendoff, 2002) in which first phonemes form words and then words (or word-phoneme combinations) form sentences. The combination of (familiar) phonemes allows the generation of a very large set of words, which can grow continuously in life. These words (including novel but phonetically regular words) can then be combined to give a practically unlimited set of sentences. Yet, it is important to realize that this two tier productivity is restricted to the languages we are familiar with. In the NBA, that means languages for which we have developed neural blackboards (van der Velde and de Kamps, 2015a).

van der Velde and de Kamps (2006, 2010) explain the structure and operations of the neural blackboards in detail. Here, we address a number of main issues, focusing on representational structures in the sentence blackboard. The composite structural elements of the sentence blackboard are “structure assemblies,” as illustrated in Figure 1. They can bind to concept assemblies (or to “word assemblies” in the phonological blackboard) and they can bind to each other to generate the structure of the sentence (e.g., *cat is on mat*).

The thick-line connections in the blackboards play a crucial role in the process of generating and representing a sentence structure. These connections are “conditional connections,” consisting of gating circuits. To operate as a connection, the gates in the connections have to be opened or activated. This ensures that activation does not flow without control in the neural blackboards, that is, the connections in the blackboards are not associative. The gates can be activated by working memory (WM) activation, representing a binding, and by control circuits, which represent (e.g., syntactic) operations in the architecture. We will discuss these operations in more detail later on.

So, the *in situ* assembly *cat* is bound (via the phonological blackboard) to a “Noun” structure assembly *Nx* in the sentence blackboard. Binding is achieved by working memory activation that opens the gates between the assemblies involved. To this end, the sentence blackboard has a number of Noun assembles which can all potentially bind to each of the Word assemblies in the phonological blackboard (via a matrix or tensor-like connection structure, see below). All bindings in all neural blackboards are of this kind. A specific binding in the “connection matrix” between assemblies is achieved by activating a specific working memory, which consists of sustained activation in a population of neurons. Once activated (by the mutual activation of the assemblies it binds), the population remains active on its own for a while due to “reverberating” activity (e.g., Amit, 1989). So, in this way, *cat* will bind to *Nx*. Similarly, *is* will bind to the Verb structure assembly *Vz*, *on* to the Preposition structure assembly *Pu* and *mat* to *Nw* (again, via the phonological blackboard).

Thus, to represent sentences based on *in situ* words (concepts), the NBA builds a connection path (structure) in the sentence (and phonological) blackboard, in accordance with the syntactic structure of the sentence. These sentences can be novel sentences based on familiar words (or even novel words based on familiar phonemes), and they can include hierarchical structures like (e.g., center) embedding (van der Velde and de Kamps, 2006, 2010). Once a connection structure is built it can be used to produce behavior, because it constitutes a connection path between the *in situ* concept assemblies it interconnects. In turn, this entails that it forms a (temporal) connection path between all perception and action structures embedded in these concept assemblies, thus forming a path between perception and action as the basis for behavior.

## 4. IDyOT: the information dynamics of thinking

### 4.1. Overview

**Figure 2:**
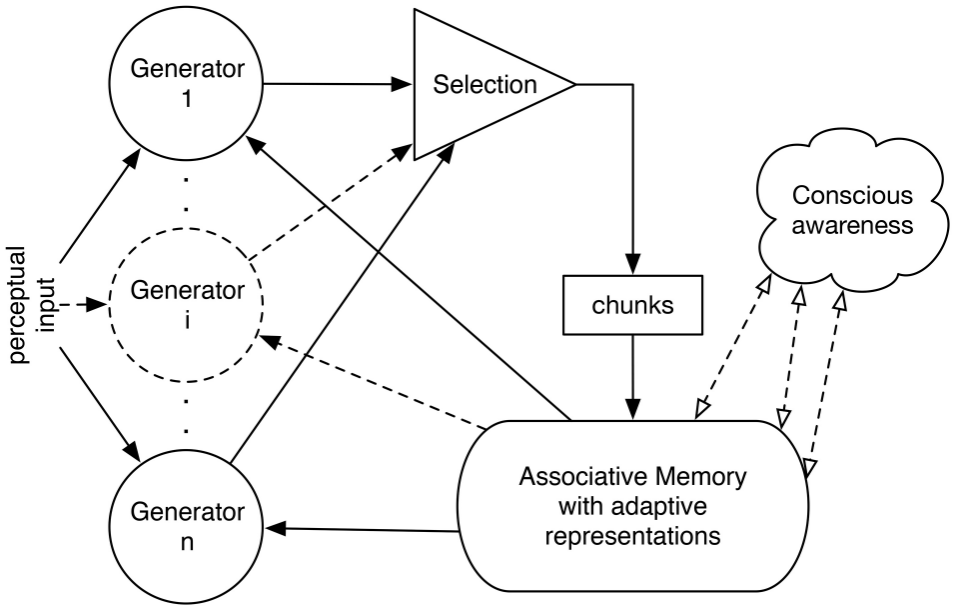
Overview of the IDyOT (Information Dynamics of Thinking) architecture. Generators synchronized to perceptual input sample, given previous perceptual input (if any), from a first-order, multidimensional Markov model to predict the next symbol in sequence, which is matched with the input. Predicted symbols that match are grouped in sequence until a chunk is detected on grounds of its information profile. The generator then stores the chunk, as described in §7.1.3 and resets its chunk, which is the sum of the structured hierarchical memory and a detector that searches for salient information, shown as “conscious awareness” here. This allows the resulting chunk of sequence to be stored in the memory, to become part of the statistical model and thence to be used subsequently.

IDyOT (Information Dynamics of Thinking: Wiggins, 2012b; Wiggins and Forth, 2015; Forth et al., 2016) implements Baars' Global Workspace Theory (GWT; Baars, 1988), affording a computational model of a hypothetical cognitive architecture. At the functional level^1^, a number of *generators* sample from a complex statistical model of sequences (explained below), performing Markovian prediction from context (Wiggins and Forth, 2015; Forth et al., 2016). Each generator indexes a string of symbols, forming a *chunk*, a final substring of the overall memory model, expressed as symbols, whose origin is explained below. Each indexed string serves as a context for prediction of the next (as yet unsensed) symbol; predictions are expressed as distributions over the alphabet used to express the input. A chunk is integrated into the memory and Global Workspace (which may be thought of as an AI blackboard: Corkill, 1991) when it meets a criterion based on information content. The upshot of this design is that IDyOT's primary cognitive operation is perceptual chunking. Figure 2 gives a functional overview.

IDyOT maintains a cognitive cycle that continually predicts what is expected next, from a statistical model, expressed in terms of self-generated symbols that are given semantics by perceptual experience; it is thus focused on sequence. Perceptual input is matched against generators' predictions, and where a match leads to a larger increase in uncertainty than other current matches, the corresponding generator's chunk is flushed into the Global Workspace, and stored in memory, linked in sequence with the previous chunk. Chunks that fail to win are forgotten after a fixed period, the duration of which is question of the research. The model entails that, for perception to work, at least some generators must be working in all perceptual modalities at all times; otherwise no generator would be predicting for input in a newly active modality to match against. This activity may account for otherwise unexplained electrical brain activity that is not directly concomitant with perceived events, and it may be responsible for spontaneous creativity (Wiggins and Bhattacharya, 2014).

### 4.2. Representation, memory, and prediction in IDyOT

Each chunk, having been recorded, is associated with a symbol in a higher-level model, which adds to the overall predictive model. Each symbol corresponds with a point in a conceptual space (Gärdenfors, 2000, 2014) associated with its own layer, and each such point corresponds with a region or subspace of the conceptual space of the layer below, defined by the lower-level symbols in the chunk. Thus, there are two parallel representations: one symbolic and explicitly sequential; and one continuous and non-sequential, but encoding sequential information. The former provides evidence from which the latter is derived, while the latter provides semantics for the former.

For grounding (or, more precisely, *tethering*: Sloman and Chappell, 2005), the lowest-level conceptual spaces are *a priori* defined by the nature of their sensory input (inspired by human biology: for example, auditory input models the output of the Organ of Corti); higher-level ones are inferred from the lower levels using the information in the sequential model. Structures may be grouped together in categories, according to similarity in their conceptual space, giving them semantics in terms of mutual interrelation. Using this, a consolidation phase allows membership of categories to be optimized, by local adjustment, in terms of the predictive accuracy of the overall model. Theoretically, the layering of models and its associated abstraction into categories can proceed arbitrary far up the constructed hierarchy (Wiggins, 2012b; Wiggins and Forth, 2015). Forth et al. (2016) provide an account of the representation of timing in IDyOT; these aspects, however, are beyond the scope of the current paper.

In general, the stimuli to which IDyOT will respond are sequences of atomic percepts. All the dimensions of music, pitch, timbre, amplitude and time, which also feature in speech, are used for prediction, as has been demonstrated in IDyOM (Pearce, 2005; Pearce et al., 2012), as can any other transduced signal. This demands a more powerful Markov model than is common in cognitive science language modeling. Conklin and Witten (1995) proposed a *viewpoint*-based approach that allows a set of interacting features, associated by means of sequences of multi-dimensional symbols, to perform multi-dimensional prediction. This is the system used in IDyOM and adapted for multidimensional language models by Wiggins (2012a). A key contribution of the viewpoint idea is the ability to superpose distributions from different features with weights determined by their entropy (Pearce et al., 2005).

Given Conklin's notion of viewpoint (Conklin and Witten, 1995) and the associated mathematics, it becomes possible also to represent propositional meaning within the statistical framework: to do this, one incorporates representations of the meaning (perhaps drawn from another sensory modality, e.g., describing in language a scene representation derived from visual input) in the statistical model (Eshghi et al., 2013). Here, we presuppose a rich, multisensory input which allows associations to be constructed between different sensory modalities, on the basis of co-occurrence.

A key scientific advantage of this representations is that its symbols are (directly or indirectly) explicable in terms of IDyOT's perceptual input, and a record of that perception is maintained. Thus, its status as a cognitive model is more easily tested than in equally powerful, but less semantically transparent, learning systems, such as deep neural networks.

### 4.3. Summary: the principles of IDyOT

In summary, the IDyOT model is based on 6 principles. Notations used in the current description of IDyOT are presented in the Table 1.

1. The fundamental function of cognition is to efficiently process sensory information so as to predict what is to happen next in the world.
2. Predictions are made by classifying events (§§6.1.3,7.1.3), counting likelihoods of short sequences, and building a literal model of the experience of the organism in these terms (§6.1.1). Predictions are expressed as distributions over alphabets of events.
3. Events are identified by chunking sensory input (§7.1.3).
4. The cognitive system always strives to maintain the optimal representation of its memory. Optimality is expressed in terms of the mean number of bits required to represent each symbol in the memory: smaller is better.
5. Meaning is constructed internally to the cognitive system, and incrementally, and consists in associations between symbols in the IDyOT memory (§§6.1.3,7.1.4).
6. Because the model maps directly to experience, it is learned incrementally (§7.1). This has the following consequences:
  - Meanings attributed to symbols depend on the order of events that the model learns (§7.1).
  - It is necessary from time to time to re-optimize the model, after an extended phase of incremental learning. This is termed *memory consolidation*. One consequence is that meanings can change retrospectively as the system learns.

**Table 1:** Notation used in the current description of IDyOT.

ℵ(*v*)	The alphabet associated with viewpoint *v*.
*D*_*t,A*_	The distribution that constitutes IDyOT's prediction at time point *t* over alphabet *A*.
*H*(*D*)	The estimated entropy of distribution *D*, over alphabet *A*: H(D) $=-\underset{s\in A}{\sum }p\left(s\right)$ log_2_ *p*(*s*).
*h*(*D, s*)	The estimated information content of symbol *s* drawn from distribution *D* over alphabet *A*: h(D,s) = −log_2_ p(s).
*S*_*A*_	The conceptual space (Gärdenfors, 2000) associated with alphabet *A*.
*R*_*A,s*_	The region of *S*_*A*_ that corresponds with the symbol *s* ∈ *A*.

## 5. NBA and IDyOT as complementary approaches to representation

Although the NBA is a neural architecture whereas IDyOT is primarily a symbolic one, they are functionally and structurally related.

In particular, chunking plays a key role in this relation between the two architectures. Perceptual chunking is the key operation of IDyOT, but it is also the underlying principle of structure formation in the NBA. The neural blackboards in the NBA not only interconnect information or provide a workspace in which information can interact and compete, they also form larger chunks of the information presented to them. These chunks arise during information processing and competition and are represented with the structure assemblies that characterize a given blackboard.

In this way, the two approaches are strongly mutually complementary: IDyOT can provide the structural elements that would be needed in a neural blackboard representation, instead of deriving them from a laborious and perhaps faulty analyses. The way in which IDyOT derives these structural elements is much more direct and secure than the engineering approach in NBA, because the elements derived by IDyOT are based on learning mechanisms using real corpora. These learning methods could also be used to develop the structural elements of a phonological neural blackboard and for neural blackboards of other languages than English.

In turn, the NBA provides a direct neural implementation of the structures as learned by IDyOT. This offers the possibilities for fast hardware implementations combined with processing abilities based on dynamic competitions in the neural blackboards. The dynamics in neural blackboards also strengthen functional processing in the architecture. For example, they can play a role in sentence processing, in the generation of behavior (e.g., answering questions) or in ambiguity resolution. They also reduce the constraints that need to be learned to perform these tasks.

In the next sections we address a number of relations between the representations used in the NBA and IDyOT in more detail.

## 6. Structural elements in neural blackboards or workspaces

The first relation between NBA and IDyOT concerns the role of neural blackboards or a workspace. In both architectures, special operators (or neural circuits) process and generate special forms of information. But to account for the productivity of human cognition there has to be a way in which the information processed or generated by special processors is interrelated and combined. A neural blackboard or workspace allows these interactions to occur, with the special processors feeding into and competing within them. The role of neural blackboards or workspace in both architectures is also related to the small-world network structures that would allow different brain processors (areas) to interconnect with each other in a flexible way.

Blackboards play a role in classical computation (Corkill, 1991), in which they allow the representation of generic forms of information that can be stored and retrieved at will (in line with the characteristics of symbolic information processing). In contrast, the neural blackboards in the NBA are not generic in this sense. They do not represent arbitrary information which can be stored and retrieved at will. Instead, the information that can be stored in a given neural blackboard is determined by the nature of its composite structural elements, which depends on the kind of process the neural blackboard is involved in. For example, the structural elements of the neural sentence blackboard are different from those in the phonological neural blackboard: the sentence neural blackboard has main assemblies and sub assemblies for specific syntactic structural elements (e,g., “clause” or “preposition”), which are not found in the phonological neural blackboard. As a consequence, the neural sentence blackboard cannot (by itself) represent phonological structures. This is why the blackboards in the NBA are referred to as *neural* blackboards, to emphasize their internal and selective neural structure.

The workspace in IDyOT is symbolic. But the composite structural elements in the workspace, learned by IDyOT, are related to the composite structural elements in the neural blackboards of the NBA.

In the NBA, however, the composite structural elements (or ‘structure assemblies’) are engineered, derived from an analysis of the domain (e.g., language) for which the neural blackboards are used. In contrast, the structural elements in IDyOT that provide a representation of phonological and sentential structures are learned from a real corpus.

It would be a huge advantage for an architecture as the NBA if the structures in neural blackboards could be learned from real corpora instead of being designed. In return, the NBA could then offer a neural (parallel and dynamic) implementation of the structures as learned by IDyOT. The following subsections illustrate, for the first time, how learning proceeds in IDyOT and how structural elements as learned in IDyOT can be implemented in a neural and dynamic manner.

### 6.1. IDyOT memory: encoding sequential structure and conceptual meaning

#### 6.1.1. Overview

Because IDyOT's learning process is incremental, as opposed to the one-shot learning of most statistical learning systems, there is diachronic development of meaning in its memory. As a result, it is difficult to see how the system works from a static, descriptive perspective. Therefore, we begin with a static description of the representation and how it is used, so that the reader has a clear idea of where the incremental process is heading. Related, but different descriptions are given by Wiggins and Forth (2015), with respect to the dynamics of lexical disambiguation, and by Forth et al. (2016) with respect to timing in music and language. First, then, the reader is asked to focus on the data structure presented, and to postpone the question of how it is constructed to §7.1. The “viewpoint” terminology used in the following description was originated by Conklin (1990) and Conklin and Witten (1995).

**Figure 3:**
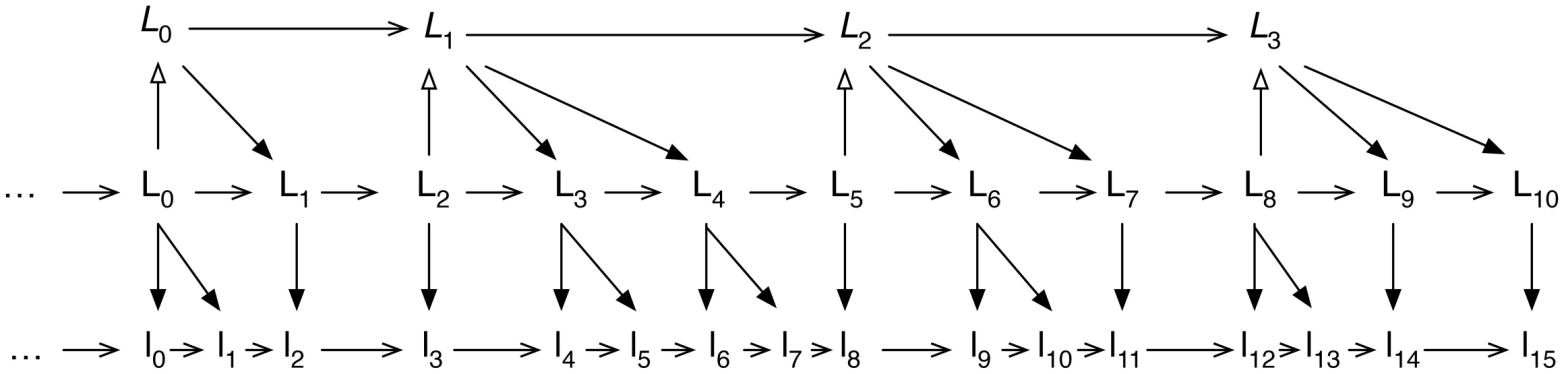
Simplified illustration of the abstract structure of IDyOT memory. The three generators are working at the level denoted by labels with upper case L; these have been derived from the lower case l labels, below, and the generators are engaged in working on the next level up, denoted by labels with upper case italic L. Arrows with empty heads denote abstraction; arrows with solid heads denote concretisation; and arrows with open heads denote temporal sequence, though note that the diagram shows only sequence, and does not represent time. Recall that each generator's chunk subtends the sequence from its pointer to the end of the memory. Finally, note that each arrow denotes a range of possible next labels, with an associated distribution, and that generators can work at any and all of these levels. The diagram is simplified by showing only one of the alternative labels that exist at each level; thus, each of the abstraction and concretisation arrows should be thought of as a range of choices, governed by a distribution derived from observed likelihood.

IDyOT's conceptual representation consists of two components, both of which are learned. The primary component is a sequence of events with separable features (*viewpoints*), annotated with chunk extents, which themselves form a sequence, and to which chunking is then applied recursively, up to a limit which is a parameter of the system (see §7.1.3); we say that a symbol at level *i subtends* a sequence at level *i* − 1; Figure 3 illustrates this. The shortest possible event is a multidimensional object that describes a simultaneous moment as sensed by IDyOT, at a sampling rate which is a parameter of the system, but of which 40 Hz is a preferred value, in terms of all the sensory modalities available to it. The examples given here are taken from auditory processing; however, there is no implication that this should be the only modality available.

#### 6.1.2. Sequence

The sequence memory consists of symbols, beginning at the lowest representational level, and recorded sequentially in perceived time, as abstractly illustrated in Figure 3. Higher layers in the hierarchy constitute abstractions of the sequences that their symbols subtend, in lower layers. Thus, once the memory is constructed, there are in general three directions of possible prediction from any given context: up, with increased abstraction, down, with decreased abstraction, and forwards in perceived sequence. The theory does not currently consider the complicating possibility of reasoning backwards, nor of subsequent conscious reinterpretation; reinterpretation should be layered on top of this. The structure so produced, combined with the contextualized distributions afforded by the transition matrices, is similar in nature to a Dynamic Bayesian Network (Pearl, 1999).

For a concrete example, consider speech input. The lowest-level representation of this would be spectral and highly granular, and therefore prohibitively expensive in memory. Since the basic symbols would, in a full example, be sensory inputs, for a human-like IDyOT, retention of the very lowest levels of memory should be fleeting, modeling echoic memory, and therefore our example is more realistic, beginning, like Wiggins and Forth (2015), at the somewhat artificial level of phonemes, pitch and amplitude: these constitute our *basic viewpoints*.

**Figure 4:**
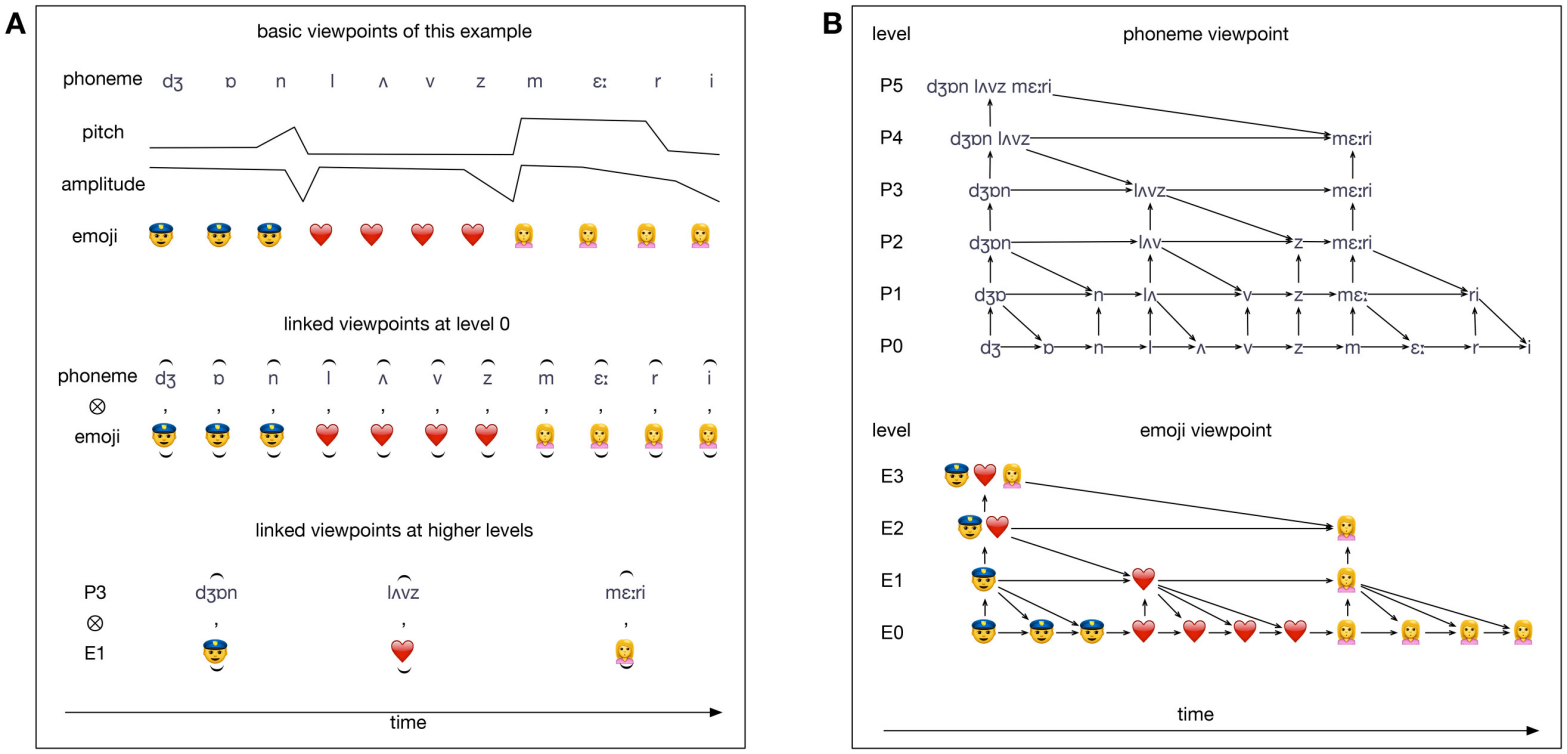
Two perspectives on IDyOT memory. **(A)** An illustration of the parallel basic viewpoints for the sentence “John loves Mary,” expressed in phonemes with associated voice pitch and amplitude signals, and semantics represented (for the purposes of example only) by emoji sequences. The top group are the *basic* viewpoints, as directly transduced (again, for the purposes of example); the middle example shows a *linked* viewpoint at the basic level; and the bottom example shows a linked viewpoint that encodes the discovered association between the semantic representations and the spoken words; such links can only take be generated when the two source viewpoints are aligned in time. **(B)** The hiearchical memory structures resulting from sensing of the sentence, “John loves Mary,” in terms of the individual phoneme and emoji viewpoints by a fully trained IDyOT. Note that this does not correspond with the standard syntactic parse, and nor is it the same as a MERGE style parse of the words. Associated with each layer of the tree, *L*, is a continuous, time-variant conceptual space, *S*_*L*_, (Gärdenfors, 2000) of timbre; this is a complex Hilbert space, whose points are time-slices in a spectral representation, such as a Fourier transform. Each stimulus at level 0 corresponds with a temporal trajectory (of variable length) in that space, while the corresponding structures at level 1 are points in a different, abstract space. *S*_*i*+1_ is related to *S*_*i*_ by spectral (e.g., Fourier) transformation, following Chella et al. (2007). Then, the sound /dZ/ is represented in full spectral detail at level 0, but in summary form, as a point, at level 1, as are /o/ and /n/. At level 1, further trajectories connect the more abstract representations, and thus the temporal detail of the individual sounds is abstracted, allowing (for example) the same word to be recognized regardless of how long the vowel takes. Expectations as to timing are generated from the various examples of each sound in each context in the memory (Forth et al., 2016).

Consider the extremely simple example sentence, “John loves Mary” in Figure 4, which illustrates the idea in multiple dimensions. For example only, we use emoji to denote the semantics of the sentence: these are presented at the same time as the example sentence is being spoken. This could be represented by viewpoint “emoji” in Figure 4A, which should be thought of as alongside the other viewpoints, together constituting level 0 in Figure 4B, simulating more complex world experience. We can consider not just single viewpoints, but also their cross products (known in Conklin's system as a *linked* viewpoint), whose alphabet consists of pairs constructed from the two source alphabets. This, of course, generates a combinatorial explosion of viewpoints.

At each layer, there is a first-order Markov model, which allows prediction of the next item in sequence; Wiggins (2012b) explains the importance of this prediction with respect to creativity. Predictions, expressed as distributions over the alphabet of the relevant layer, may be generated for any point at the leading edge of the hierarchical memory structure as it is generated: thus, higher-level, abstract predictions are current at the same time as surface-level ones, and this is how long-term dependency in language, music, and narrative is managed.

#### 6.1.3. Meaning

IDyOT is unusual as a symbolic learning system because it does not use symbols with predefined meanings. Rather, symbols are grounded in perception, and their meaning is determined either in terms of synchronic relations between sensory modalities, or in terms of the diachronic sequence chunks that they subtend. In either case, meaning is placed in context of the conceptual spaces (Gärdenfors, 2000, 2014) associated with the viewpoints and the alphabets built above them. To summarize very briefly, conceptual spaces are low-dimensional geometrical spaces that afford judgments of similarity or betweenness. An example is the familiar color spindle, which has regions corresponding with colors of the spectrum, in which Euclidean distance models similarity (Gärdenfors, 2000, 2014). Different perceptual phenomena exhibit different geometries (for example, musical pitch is a spiral, Shepard, 1964), and methods for deriving these properties are a rich area of future research; Tenenbaum et al. (2011) propose various candidate statistical structures. In the higher layers of IDyOT memory, because a symbol subtends a sequence of symbols below it, it must be possible to map a trajectory of points or regions in a lower space to a single point in a higher one; this suggests that spectral representations are a promising route; Chella et al. (2008) and Chella (2015) suggest methods.

The conceptual spaces in IDyOT are important, because they afford the similarity measures that categorize chunks together in the incremental chunking and representation process, which we describe in §7.1.

### 6.2. NBA: binding sequential structures and concepts

The abstract structure of IDyOT memory, as illustrated in Figure 3, consist of learned components, organized in hierarchical layers. They form the link between the learning mechanisms of IDyOT and the neural blackboard structures of the NBA.

**Figure 5:**
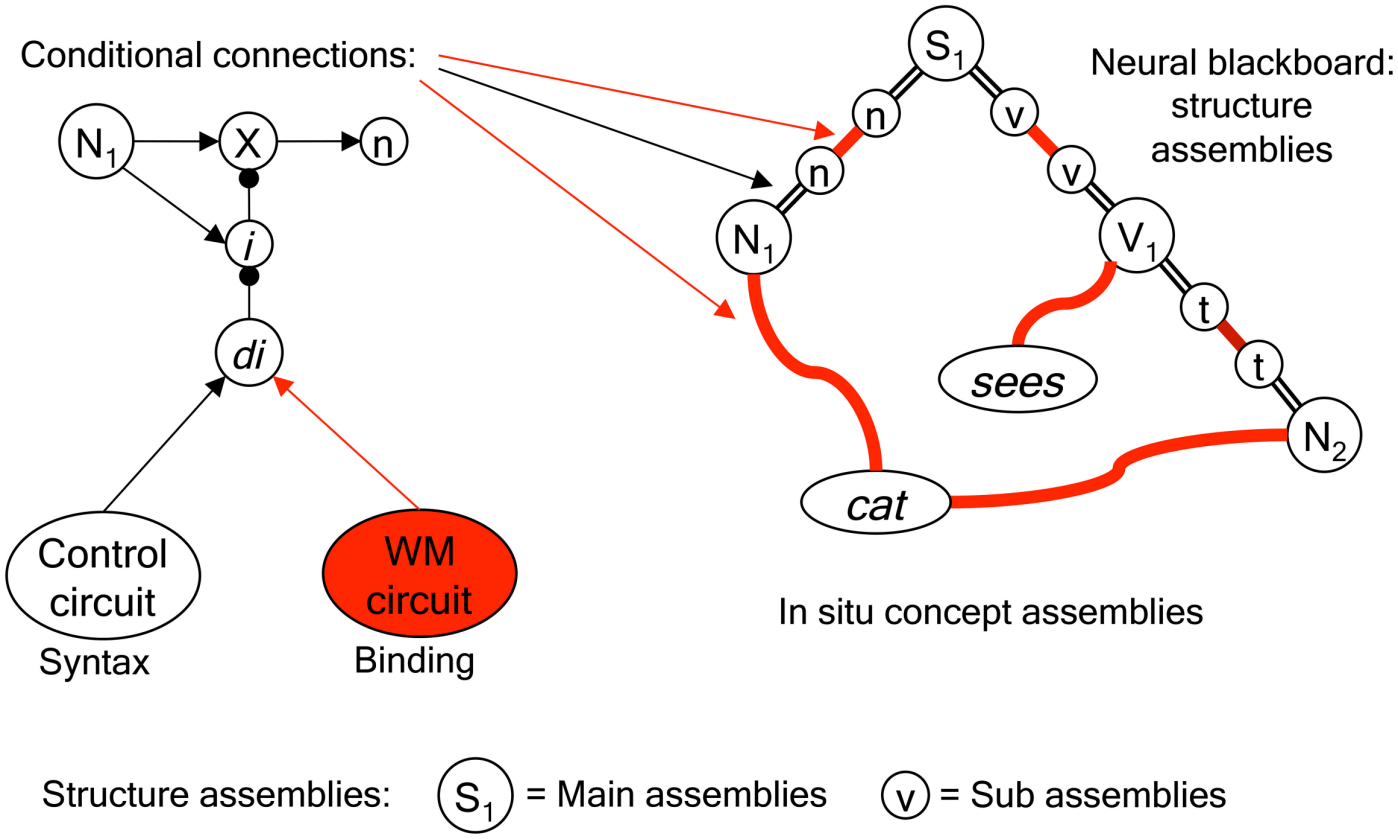
**Left**: conditional connections. N, n, noun; *i*, inhibition; *di*, dis-inhibition; WM, working memory. **Right**: Representation of *cat sees cat* in the sentence neural blackboard. S, sentence; V, v, verb; t, theme (object).

Figure 5 illustrates these neural blackboard structures in more detail, with the structure the sentence *cat sees cat*, to compare the encoding of sequential structures in IDyOT and the NBA.

The red and black thick lines in the figure illustrate the (crucial) conditional connections in the NBA, which consist of gating circuits. In the NBA, each concept assembly (e.g., of a noun) is connected to a set of structure assemblies of the same kind (all *Ni* assemblies in the case of a noun) with gating circuits. (In fact, the words need to be represented in a phonological blackboard first, to enhance the productivity of the architecture, being able to represent novel but phonologically regular words, and to reduce the number of conditional connections in the architecture.) In turn, each structure assembly consists of a “main assembly,” such as *N*1, and (a set of) sub assemblies, such as *n* or *t*. The connection between a main assembly and a sub assembly consists of a gating circuit as well.

Structure assemblies of different kinds, such as *V*1 and *N*2, are connected by their sub assemblies of the same kind. Here, by their *t* (theme) sub assemblies, which represents the fact that a verb can have a theme (object). This connection (red line) also consists of a gating circuit, which can be activated by a WM neural population. This results in the binding of the two connected sub assemblies and hence their main assemblies, which last as long as this WM population is active. When two sub assemblies are bound in this way, activation can flow from one of the main assemblies to the other, by opening the gates between these main assemblies and their sub assemblies.

The gating circuits operate by disinhibition (*di*), as illustrated in Figure 5. When *N*1 is active, it activates a neuron (or neuron population) *X* and an inhibitory neuron (or population) *i*. The latter inhibits *X*, which blocks the flow of activation. But when *i* itself is inhibited (by neuron or population *di*), activation can flow from *N*1 (via *X*) to *n*.

Gating circuits can be disinhibited (or “activated”) in one of two different ways. In the case of gating circuits between main assemblies and sub assemblies (the black connections in Figure 5), the activation results from an external control circuit that activates the *di* population. This is how syntactical operations affect binding in the blackboard. A control circuit could have recognized that *sees cat* represent a verb and a theme. It then activates all *di* populations in the gating circuits between all *Vi* and *Nj* assemblies and their *t* assemblies. As a result, the active *Vi* and *Nj* will activate their *t* sub assembly.

**Figure 6:**
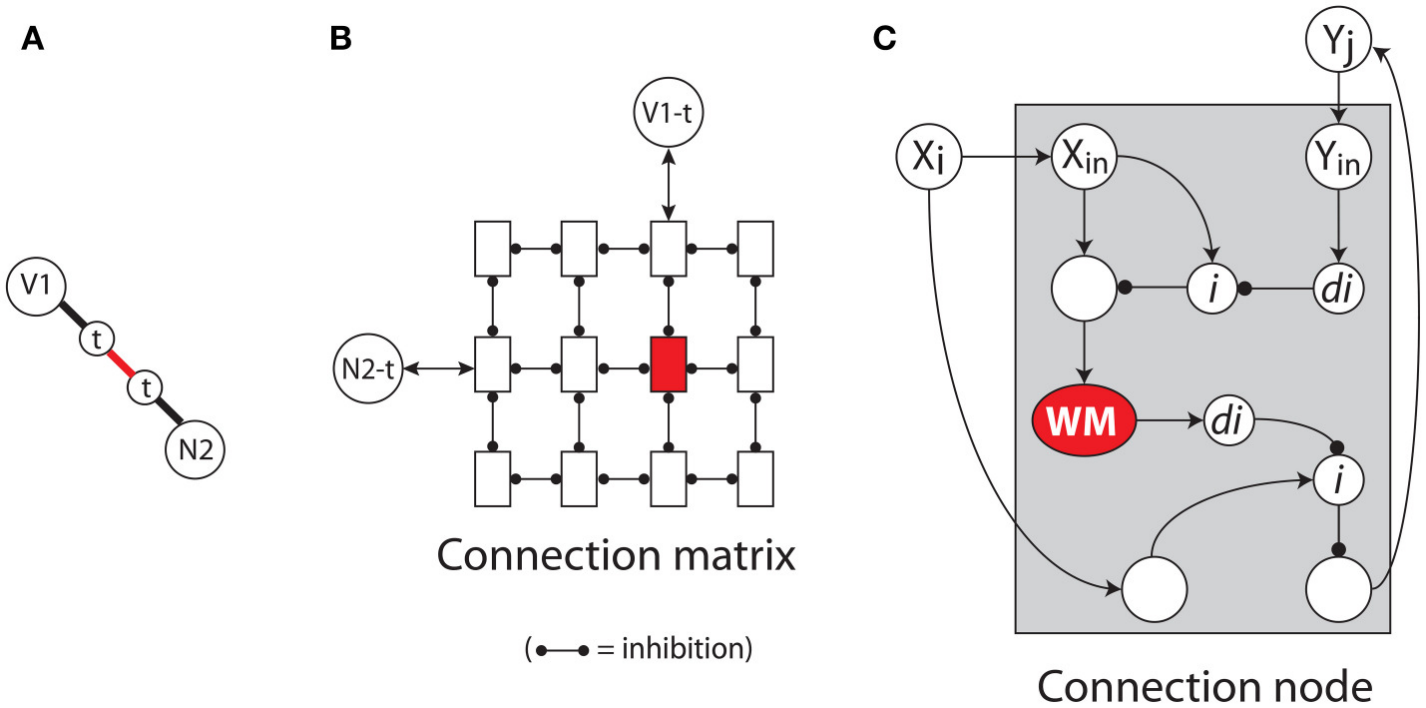
**(A)** Conditional connections. N, noun; V, verb, t, theme (object). **(B)** Connection matrix. **(C)** Connection node. *i*, inhibition; *di*, dis-inhibition; WM, working memory.

Gating circuits between sub assemblies and between word and main assemblies (the red connections in Figure 5) are activated by (specific) “working memory” (WM) populations. A WM population remains active for a while, after initial activation, by reverberating activation in the population (e.g., Amit, 1989). An active WM population binds the assemblies to which it is connected. Figure 6 illustrates how this is achieved in the NBA. Figures 6A–C illustrate the same binding process with increasing detail. In Figure 6A, the binding between the *t* sub assemblies of *V*1 (or *V*1−*t*) and *N*2 (*N*2−*t*) in Figure 5 is repeated. Figure 6B illustrates that this binding is based on a “connection matrix,” which consists of columns and rows of “connection nodes,” which are illustrated in Figure 6C.

Each specific *Vi*−*t* and *Nj*−*t* pair of sub assemblies is interconnected in a specific connection node, located in a connection matrix dedicated to binding *Vi*−*t* and *Nj*−*t* sub assemblies. In general, when two assemblies *Xi* and *Yj* (e.g., *Vi*−*t* and *Nj*−*t*) are concurrently active in the processing of a sentence, they activate a WM population in their connection node by means of a gating circuit, as illustrated in Figure 6C. In turn, the active WM population disinhibits a gating circuit by which activation can flow from *Xi* to *Yj*, and another such circuit, not show in (c), by which activation can flow from *Yj* to *Xi*. As long as their WM population is active, *Xi* and *Yj* are “bound” because activation will flow from one to the other whenever one of them is (initially) activated.

The NBA allows any noun to bind to any verb in any thematic role using dedicated connection matrices. Also, the NBA has structure assemblies that can bind to other structure assemblies, such as *S*1 in Figure 5 or clause structure assemblies. In this way, hierarchical sentence structures can be represented, such as relative or complement clauses.

#### 6.2.1. Sentence structure as connection path

To form a sentence structure, the structure assemblies have to bind to each other. This process is regulated by control circuits that build a sentence structure in line with the (syntactical) relations in the sentence (van der Velde and de Kamps, 2010). So, with *cat sees cat* in Figure 5, the control circuits will recognize *cat* as the subject of the sentence, expressed by the binding of *N*1 to the ‘Sentence’ structure assembly *S*1, and *sees* as the verb of the main clause, expressed by binding *V*1 with *S*1.

But then, the control circuits will recognize the second occurrence of *cat* as the object of the sentence. This seems to pose a problem, because that would seem to require a copy (different token) of *cat* to bind as the object to the verb. Indeed, symbol manipulation represents the sentence *cat sees cat* with two tokens of *cat*. But in the NBA, a given concept assembly can bind to different structure assemblies at the same time, allowing the creation of sentence structures in which words are repeated, as illustrated in Figure 5. However, the concept assemblies remain *in situ* in this way, so words in sentence structures are always content addressable and grounded. This example illustrates how the NBA solves the “problem of two” posed by Jackendoff (2002).

The sentence structures in the NBA (as illustrated in Figures 1, 5) and IDyOT (e.g., *John loves Mary* in Figure 4) are structurally similar. The sentence in IDyOT is derived from its learning principles, as outlined above, and it can be represented in the NBA in the manner illustrated in Figure 5.

As we argued, the representational similarities between IDyOT and NBA would offer a basis for combining the learning mechanisms of IDyOT, based on real corpora, with the parallel and dynamic implementation of the NBA. The dynamics in the neural blackboard can in fact be used to solve forms of (e.g., sentence) ambiguity (van der Velde and de Kamps, 2015b), which in turn offers the possibility of further reduction of the constraints that would have to be learned to represent and process complex cognitive structures.

## 7. Processing of sequential structures

A second link between the NBA and IDyOT concerns the processing of sequential information. Based on its learning mechanism, IDyOT derives probabilistic choices between structural interpretations of the processed information, in the form of transition matrices. Based on learning, predictions can be made that influence further processing of the input sequence.

The NBA uses similar kinds of information to train control circuits that selectively activate the neural blackboards, as illustrated in Figure 5. Control circuits have been implemented with feedforward networks (van der Velde and de Kamps, 2010) and, more recently, with reservoirs (Jaeger and Haas, 2004) consisting of “sequence nodes” (van der Velde, 2016a).

Similar to the connection nodes in Figure 6, each sequence node has a column structure with gating circuits that control the activation of the node. This activation depends on three sources: previously activated sequence nodes (hence forming a chain of nodes in the reservoir, representing sequential order), external activation generated by the (ongoing) input sequence, and activation already generated in the neural blackboard. The latter includes the predictions generated in the neural blackboard in the course of processing an input sequence, as in the resolution of ambiguity (van der Velde and de Kamps, 2015b).

The reservoir can, for example, learn to answer the question *Where is cat?* with the sentence *Cat is on mat* in Figure 1. The reservoir can learn to do this by recognizing the sequence *Where - localizer - noun - Agent*. Here, the sequence *Where - localizer - noun* is based on transforming the question *Where is cat?* in a more general form (with *is* = *localizer* and *cat* = *noun*). The *Agent* in the sequence is derived from the activation of the neural blackboard representation of *cat is on mat*, because *cat* in the question *Where is cat?* activates its *in situ* neural assembly (Figure 1) and thus the part of the neural blackboard representation of *cat is on mat* to which the assembly *cat* is bound. In this way, the reservoir can learn to reactivate the sentence representation of *cat is on mat* in the neural blackboard, to generate the answer *mat* (van der Velde, 2016b).

But, for example, the transformation of the question *Where is cat?* into the more general form *Where - localizer - noun?*, learned by the reservoir in the NBA, is based on an analysis. In contrast, the learning mechanism of IDyOT can provide the information to train the reservoir in the NBA, based on real corpora. Conversely, the distinction between structured neural blackboards and the control reservoirs in the NBA can strongly reduce the number of contingencies that have to be learned over time, as illustrated with the ease with which the reservoir can learn to recognize *Where - localizer - noun - Agent* (van der Velde, 2016b).

The more elaborate learning mechanism of IDyOT would thus have to be integrated with the NBA, and eventually be implemented with neural reservoirs that interact with the neural blackboard in the NBA. The learning process in IDyOT is outlined in more detail below, again for the first time.

### 7.1. The IDyOT incremental learning process

#### 7.1.1. Initial state

Initially, IDyOT has no memory, no symbols, and only inputs. Input is in terms of percepts conceptualized as symbols representing continuous real-world phenomena at whatever level of abstract is chosen: here, phonemes, pitch, amplitude, and observed meaning (emoji).

#### 7.1.2. Chunks and labels

**Figure 7:**
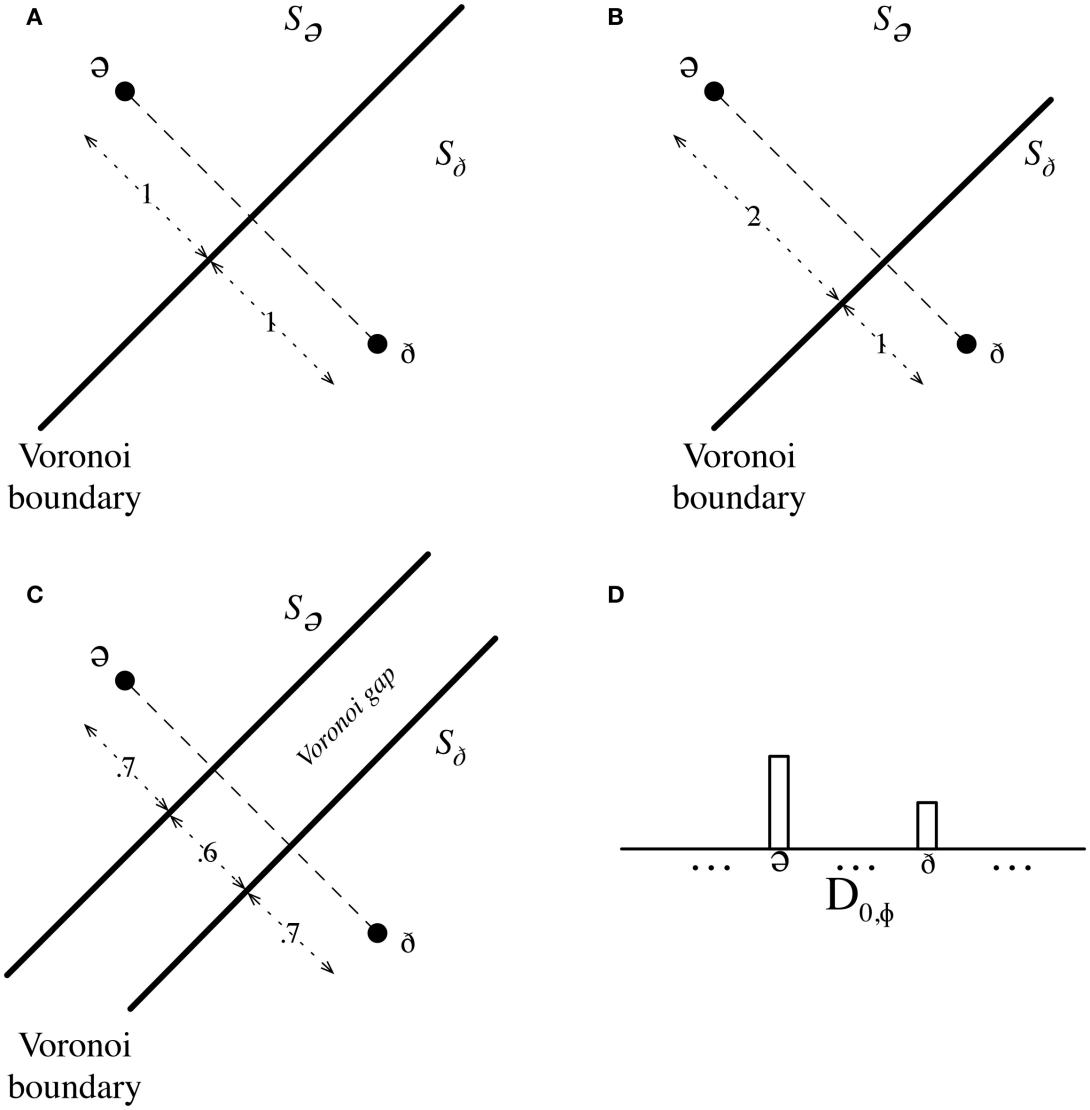
Modulating conceptual regions according to expectedness. **(A)** The unmodified Voronoi tessellation of the conceptual space of phonemes, *S*_Φ_, showing the boundary between *R*_*S*_Φ,ә__ and *R*_*S*_Φ,ð__. **(B)** The modified tessellation; note that the distances from the labeled points to the boundary have changed in proportion to the relative likelihoods in *D*_0,Φ_. **(C)** The tessellation with a non-zero gap. **(D)** Schematic partial representation of the distribution, *D*_0,Φ_ showing (imaginary) proportions for /ә/ and /ð/.

Given a low-level, predefined conceptual space, *S*_*v*_ (which initially has no geometry, but learns it as more data is received), for each low-level viewpoint, *v*, IDyOT labels the mutually discriminable points in *S*_*v*_ with symbols, building an alphabet, ℵ(*v*), and, separately, builds a chain of these symbols as the input sequence proceeds; this may be thought of as the chain l_*i*_ in Figure 3. Simultaneously, IDyOT builds a first order transition matrix of the chain; this will allow the construction of successive distributions over ℵ(*v*), *D*_*t*, ℵ(*v*)_, as time, *t*, proceeds. Each symbol is considered in relation to the symbols already created, in terms of their corresponding points: a quasi-Euclidean distance (*norm*), in *S*_*v*_, may be computed between them. At the same time, the space is progressively partitioned into regions whose points are nearest to each point in the sequence, as in a Voronoi Tessellation (Aurenhammer, 1991). This tessellation, possibly modified by a parameter which creates a gap between the regions (Figure 7), forms the basis of similarity comparison. Points in (non-zero) gap regions form new seeds. This process will, of course, produce initially inaccurate predictions and labelings, but as sufficient data is processed, these early errors fade into statistical obscurity, propelled by the memory consolidation process described below.

However, this simple mechanism would not account for the human propensity to perceive what is expected, because *S*_*v*_, the conceptual space associated with *v*, is static. The distribution, *D*_0,ℵ(*v*)_, describes IDyOT's expectation at this point; it is derived from the transition matrix for *v*. Each region, *R*_*v,s*_, where *s* ∈ ℵ(*v*) in the Voronoi tessellation of *S*_*v*_ is now expanded or contracted, by changing the position of each plane dividing the space, in proportion to the relative likelihood of the symbols corresponding with the points to whose connecting line the plane is perpendicular. A parameter, whose value is the subject of study, determines the degree of variation; an interesting possibility is that this value is related to entropy of the distribution, as was found empirically to be case in a related application of distributions in IDyOM (Pearce et al., 2005), where distributions containing more information influence the outcome more. Thus, the less expected a phoneme, *s*, is, the smaller its *R*_*v,s*_ temporarily becomes, and so a phoneme that is both imprecisely articulated and unexpected may be misidentified as one near it, which is more likely in the distribution (Figure 7). IDyOT behaves like a human in this context: it commits to memory incorrect perceptions, as if they were correct.

#### 7.1.3. Chunking: competition and boundary entropy

Each new symbol, indexing a point in *S*_*v*_, is available to all generators associated with this viewpoint (see Figure 3). As the transition matrix is populated, predictions can be made of likelihood, and as IDyOT's memory develops, progressively more informed predictions may be made using the probabilistic network afforded by the layered memory. Thus, the entire context will influence *D*_*t*, ℵ(*v*)_ it any time point *t*. Again, initially, these predictions will not be particularly accurate; as more data is received they will improve. As each new label appears, therefore, a new distribution is generated, and its entropy, *H*(*D*_*t*+1,ℵ(*v*)_) can be calculated and compared with *H*(*D*_*t*,ℵ(*v*)_). On the basis of empirical evidence from computational linguistics and music cognition (e.g., Sproat et al., 1994; Brent, 1999; Pearce et al., 2010; Rohrmeier et al., 2015), at each time step, IDyOT's agents compete for global workspace access, the largest positive change being the winner. If no agent registers an increase in entropy, there is no winner, and no change in the memory; IDyOT proceeds to the next input stimulus.

Thus, IDyOT achieves hierarchical perceptual chunking.

#### 7.1.4. Layer formation and abstraction

Following the identification of a chunk in memory, IDyOT must decide whether to generate a new label or to label this chunk with an existing symbol, on grounds of similarity. In the former case, a new label is generated, at level L_*i*_ in Figure 3, and it is added to memory, along with pointers to the lower level chunk that it subtends; also, the transition matrix for the upper layer is updated. A further transition matrix, of which one exists for each pair of contiguous levels, is also updated with the new symbol and transition. In this connection, a higher-level symbol is deemed to connect down to any symbol in its chunk, while any lower level symbol is deemed to connect to any symbol in whose chunk it appears. It is implicit in this process that each symbol in an IDyOT memory chain may be subtended by more than one symbol at the immediately higher level, and it may subtend more than one symbol below. Transition matrices for these upward connections, too, must be maintained.

Returning to the example: the higher level sequence has a transition matrix, and so its entropy can be determined, symbol-wise, as above, and therefore the same boundary test as above can be applied. If a new chunk at this level is detected, then the same process applies, and so on up the layers of the network, using the same principle of similarity measurement as above. This first generates level *L*_*i*_ in Figure 3, and then on beyond the scope of that simple example.

This recursive process constructs a tree from the very lowest level of representation up to the highest possible abstraction, as shown for our concrete example, in Figure 4. Although this simple example has focused on only one aspect of the stimulus, it is important to recall that, in a fully implemented IDyOT, all modalities of perception would be active simultaneously, and synchronized (Forth et al., 2016) in such a way as to interrelate simultaneous stimuli. Thus, the association between, for example, the word “orange”, the sound [6rInZ], and appropriate representations of the corresponding color, fruit, pop star and politics, could be learned, as illustrated in Figure 4.

## 8. Further relations between NBA and IDyOT

### 8.1. Conceptual spaces

The semantics underlying the IDyOT and NBA representations are derived from the conceptual spaces with which they interact. In turn, the conceptual spaces play a role in processing in both architectures. For IDyOT, the role of conceptual spaces is illustrated in Figure 4. In the NBA, representations of conceptual structures (relations, propositions, sentences) are based on content addressable concept representations, which directly and selectively activate conceptual structures in neural blackboards. Also, conceptual domains and relations are needed to influence sequential processing in the control reservoirs of the NBA (van der Velde, 2016a).

McGregor et al. (2015) outline a basis for a geometrical conceptual space, with interpretable spaces and dimensions derived from observed co-occurrence statistics in a large corpus. Conceptual relations and domains can be obtained by the techniques described by McGregor et al. (2015) and by the metric based on a semantic map as derived by van der Velde et al. (2015). This semantic map also consists of a co-occurrence matrix, derived from human categorizations. The metric provided a similar concept-cluster structure as derived from reduction techniques. But it also revealed the possibility of deriving bridges between conceptual domains based on metric violations.

The geometrical nature of such a conceptual space provides a natural representation for the content addressable concept representations underling the combined IDyOT-NBA architecture. Furthermore, the geometrical nature of this conceptual space and the neural blackboard mechanisms of the IDyOT-NBA architecture provide the possibilities of new forms of hardware implementations that can circumvent the limitations of the Von Neumann Architecture, on which symbolic computation is standardly based.

### 8.2. Brain and computation

As referred to in our introduction, the processing of conceptual structures can be studied with the aim to understand human cognition and its relation to the brain. Or they can be targets for the development of artificial cognitive systems. We argue that a combined IDyOT-NBA architecture can address both aims.

Because learning in IDyOT is based on information found in real corpora, it derives structures and processes based on human information processing and generation. In this way, the NBA structures and processes derived from IDyOT will be based on human information processing as well. The neural implementation of the NBA then allows a comparison between the structures and processes of the combined architecture with those observed in brain research.

**Figure 8:**
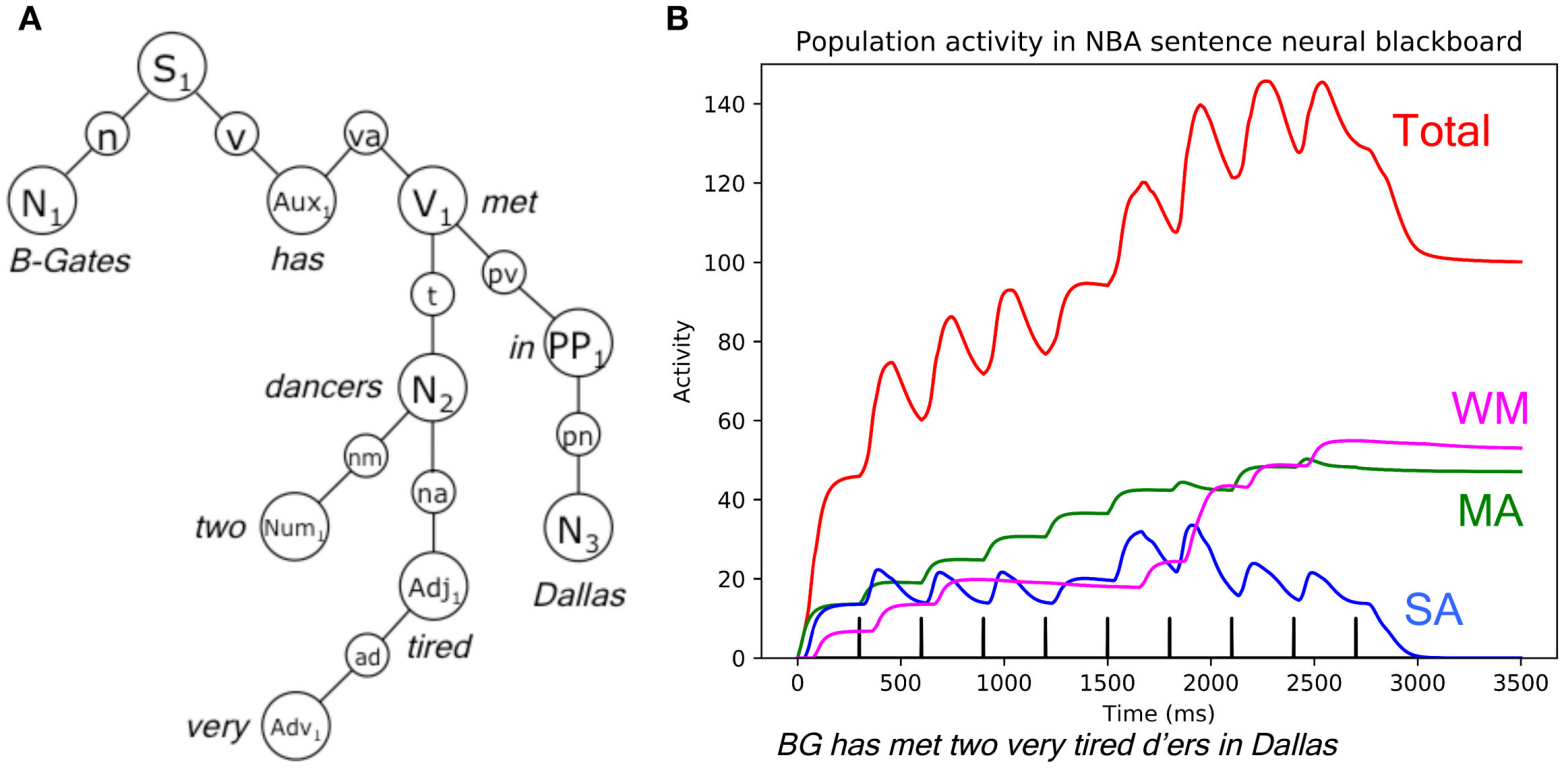
**(A)**: NBA structure of *Bill-Gates has met two very tired dancers in Dallas*, with *B(ill)-Gates* as one noun. Aux, va, auxiliary verb; Adj, na, adjective; Adv, ad, adverb; N, n, noun; Num, nm, numerator; PP, pv, pn, preposition; S, sentence; V, v, verb. **(B)**: Neural activity in the NBA when *Bill-Gates has met two very tired dancers in Dallas* is processed. BG, Bill Gates; d'ers, dancers; MA, main assemblies; SA, sub assemblies; WM, working memory. ‘Total’ (red activity) is the sum of the activation of all neural populations in the NBA structure of this sentence (over 300 populations), simulated with Wilson-Cowan population dynamics. The words of the sentence are presented at the times indicated with the vertical bars. The last bar signals the end of input activation.

An example of how the combined architecture can be related to neuro-cognitive processing is presented in Figure 8. The figure illustrates a novel simulation of NBA activity, with the processing of the sentence *Bill-Gates has met two very tired dancers in Dallas*, with *Bill-Gates* as one noun (BG). Activation of “main assemblies” (MA), “sub assemblies” (SA) and binding in working memory (WM) are shown, because they determine the representation structure of the sentence in the sentence neural blackboard of the NBA (van der Velde and de Kamps, 2006). Also shown is the overall activation of all assemblies and circuits, consisting of more than 300 neural populations in all (marked “Total”; red line). The neural populations are simulated with Wilson and Cowan population dynamics (Wilson and Cowan, 1972).

Using intracranial measurements, Nelson et al. (2014) observed that binding of words and phrases produces an increase and then decrease of activity (e.g., because binding related activation will reduce after binding). The NBA activation simulates this effect, and also indicates why it occurs, i.e., which structures and processes are related to this effect. In particular, total neural activity first increases when a new word is presented (as illustrated by the increase of total activity at the location of the black vertical bars, that indicate the presentation times of the words). But then, total activity drops, due to the binding of the presented word to previously presented words and phrases in the developing sentence structure in the sentence neural blackboard of the NBA. Occasionally, activity does not decline, as with *Bill has* or *very tired*, which results from the fact that *Bill* is the first word, which cannot bind to other words yet, and *very* does not bind to the previous word *two*.

Hence, the simulation illustrates the close relation between neural dynamics and the representation structures underlying processed sentences in the NBA. The aim of the integration of NBA with IDyOT is to develop these representation structures by learning from real corpora. In this way, machine learning could be related to brain activity observed in human cognitive processing.

Furthermore, the NBA predicts the existence of “connection” fields (or matrices) with special roles, such as “agent” and “theme” (object) in which bindings between (e.g.,) arbitrary verbs and nouns as (agent or theme) arguments can occur. Recent fMRI observations indicated the existence of (agent and theme) areas in the cortex that are selectively activated when nouns function, respectively, as agents or themes of verbs (Frankland and Greene, 2015). The activation patterns in these areas also concur with the activation patterns produced in the NBA. These areas could form a neural substrate for (parts of) a Global Workspace, in which competitions between neural structural representations could occur.

The combined IDyOT-NBA architecture also targets the development of artificial cognitive systems. Recently, Lake et al. (2016) argued that, despite recent successes, Deep Learning does not capture essential characteristics of human learning and processing. One of the difficulties for Deep Learning concerns compositional (combinatorial) processing, in which structured information is processed in terms of already familiar constituents and partial structures.

A crucial feature of compositional processing is the interaction between specialized processors and domains in which these processors, and the information they process, can interact, compete, and be combined. This is what the neural blackboards and the workspace in NBA and IDyOT are about. Because the combined architecture can develop and activate these structures based on learning from real corpora, it can address key features of human cognitive processing.

The combined architecture can also address new demands on computing power because the NBA can be implemented fully as a system operating in parallel, based on dynamic interactions. Of course, processing will be sequential when input is presented in a sequential manner. Also, the dynamic interactions will proceed in time as well. But each of the components (e.g., connection nodes in the connection matrices) will operate in parallel with all other components, and their interactions are based on direct dynamical activation and competition. When implemented in hardware, this allows the system to operate at minimal levels of power, with fast processing speeds.

## 9. Conclusion

We have presented two knowledge representations, used in two cognitive architectures, the NBA and IDyOT, that both aim to account for conceptual representation and processing in productive forms of cognition. Although the architectures differ in that the NBA is neural and IDyOT is symbolic, they are also similar in many ways. Both assume that conceptual representations consist of structures in which all aspects related to a concept are interconnected. Both assume that processing with representations occur in blackboards or a workspace, in which these representations can interact and can be (re)combined. And both rely on the principles of chunking to generate higher-level structural representations based on the more elementary ones.

Finally, the relations between both architectures combined with their different bases provide unique opportunities for a complementary integration. The NBA could provide a neural implementation of the processing and representation of higher level conceptual representations and IDyOT could provide the learning mechanisms by which the more elementary representations needed for this implementation could be derived from human cognitive (corpus) material.

## Author contributions

FvV and DN wrote the sections on NBA. GW wrote the sections on IDyOT, based on discussions with JF. The rest was a joint effort.

### Conflict of interest statement

The authors declare that the research was conducted in the absence of any commercial or financial relationships that could be construed as a potential conflict of interest.

## Footnotes

^1^The formal implementation of this functional behavior is somewhat different in actuality. However, the description given here is easier to understand in isolation.
